# Contribution of migrant coffee labourers infected with *Onchocerca volvulus *to the maintenance of the microfilarial reservoir in an ivermectin-treated area of Mexico

**DOI:** 10.1186/1475-2883-6-16

**Published:** 2007-12-18

**Authors:** Mario A Rodríguez-Pérez, Aldo Segura Cabrera, Cristian Lizarazo Ortega, María-Gloria Basáñez, John B Davies

**Affiliations:** 1 Centro de Biotecnología Genómica, Instituto Politécnico Nacional, Blvd. del Maestro esquina Elías Piña, Col. Narciso Mendoza, 88710, Reynosa, Tamaulipas, México; 2 Department of Infectious Disease Epidemiology, Faculty of Medicine (St. Mary's campus), Imperial College London, Norfolk Place, London W2 1PG, UK; 3 Department of Parasite and Vector Biology, Liverpool School of Tropical Medicine, Pembroke Place Liverpool, L3 5QA, UK

## Abstract

**Background:**

Since 1991, in Mexico, ivermectin has been administered twice a year to all residents in the onchocerciasis endemic foci which are mainly located in the coffee growing areas. However, the presence of a potentially infected itinerant seasonal labour force which is not treated regularly could jeopardise the attainment of the 85% coverage which is the present target for elimination of the disease.

**Methods:**

The prevalence and intensity of *Onchocerca volvulus *microfilariae (mf), as well as their transmission from humans to vectors, were assessed during the coffee planting-clearing and harvesting seasons of 1997–1998, and 1998–1999 in two localities (I and II) of Southern Chiapas, Mexico, which regularly receive an influx of untreated migrant coffee labourers.

**Results:**

Localities I and II had, respectively, an average of 391 (± 32) and 358 (± 14) resident inhabitants, and 70 (± 52) and 498 (± 289) temporary labourers. The ratio of migrants to residents ranged from 0.1:1 in locality I to 2.4:1 in locality II. The proportion of infected *Simulium ochraceum s.l*. parous flies was significantly lower in locality I than in locality II, and significantly higher during the stay of the migrants than before their arrival or after their departure. Parity and infection were higher in May-July than in November-February (in contrast with the latter being typically considered as the peak onchocerciasis transmission season by *S. ochraceum s.l*.).

**Conclusion:**

The presence of significant numbers of untreated and potentially infected migrants may contribute to ongoing transmission, and their incorporation into ivermectin programmes should be beneficial for the attainment of the elimination goals of the regional initiative. However, the possibility that the results also reflect transmission patterns for the area cannot be excluded and these should be analyzed further.

## Background

Human infection with *Onchocerca volvulus *still constitutes an important public health problem despite resounding control achievements in some areas of West Africa and the Americas, with a recent estimate indicating that at least 37 million people remain infected, mostly in Africa [1]. In the Americas, the presence of infected migrant labourers who fail to receive regular ivermectin treatments may play a significant role in maintaining the infection reservoir, jeopardizing the goal of eventual parasite elimination from the region. However, the impact of such temporary influx of migrants has seldom been ascertained (but see ref. [2]).

In Mexico, the main onchocerciasis-endemic focus is situated in the Southern state (Soconusco) of Chiapas, which is contiguous with the Northwest Guatemalan (Huehuetenango) endemic focus forming a single endemic region [3]. Here, *Onchocerca volvulus *is transmitted mainly by *Simulium ochraceum sensu lato *[4]. Overall, the Mexican foci have over 25,000 cases [5]. The Onchocerciasis Elimination Program for the Americas (OEPA) has eliminated severe pathological manifestations of the disease and reduced morbidity [6] through mass distribution of ivermectin (Mectizan^®^), a safe drug that kills microfilariae (mf). Adult worms are not immediately affected, but repeated exposure to ivermectin affects both the fertility and survival of adult worms [7]. As OEPA has also made progress towards its ultimate goal of eliminating the infection in several foci of the region [8-13], there is also hope that if the human microfilarial load can be kept below its breakpoint density (a level not yet determined), transmission may be interrupted and the parasite reservoir eventually eliminated [14]. Unfortunately, when this study was completed the prospects of transmission interruption in Mexico had been less successful than expected (given the low competence of the main vector at low microfilaridermia levels [15]) despite high levels of coverage and compliance to multiple biannual ivermectin treatments [10,16].

The factors that could be involved in maintaining transmission are multiple. It has been argued that migrant labourers that cross the Mexican-Guatemalan border may spread the infection within the endemic foci [5,17]. In this area, onchocerciasis is associated with coffee plantations (locally known as 'fincas') and the seasonal transmission peaks (according to entomological studies conducted mainly in Guatemala) coincide with the timing of coffee harvest [18-20]. It has been suggested that the origin of the onchocerciasis endemic focus in Chiapas was a consequence of the migration of labourers from Guatemala as the cultivation of coffee extended to Southern Mexico, and that the Northern Chiapas focus was established because of annual visits of workers to the Southern Chiapas focus for the coffee harvest [20,21]. (Presently, little migration from the Northern to the Southern Chiapas focus for coffee harvesting is observed.) As migrant labourers moving between endemic and non-endemic areas are, in general, left outside of the treatment schemes, it has been hypothesised that a significant number of untreated migrants may contribute to the parasite pool for microfilarial transmission [16]. In order to evaluate this hypothesis, the aim of this study was to estimate the prevalence and intensity of microfilarial infection in both resident and migrant populations of, respectively, stable villages and adjacent coffee fincas as well as the prevalence of infection in parous (surviving) flies sampled from the host-seeking vector population at these localities, in order to ascertain the influence on the transmission from humans to vectors of migrant, untreated workers. Since migration patterns vary locally and seasonally, the study included parasitological and entomological surveys during the planting-clearing and harvesting seasons of 1997–1998 and 1998–1999.

## Methods

### Description of the history of treatment and of the study area

The onchocerciasis control programme in Mexico began treatment with ivermectin in 1989, initially providing treatment only to patients positive at nodulectomy and/or presenting with Mazzotti reaction (to oral diethylcarbamazine) who were residents from hyperendemic villages (microfilarial prevalence ≥ 60%). From 1991 to 1994, bi-annual treatment with ivermectin was extended to all eligible residents of mesoendemic villages (microfilarial prevalence between 20% and 59%), and to 25% of those residing in hypoendemic villages (microfilarial prevalence < 20%). From 1995 to 1997, the coverage in hypoendemic villages increased to 40% of all eligible residents. Since 1997, the national strategy has been to provide mass biannual treatments to every eligible resident from all the at-risk villages (from hypo- to hyperendemic villages), and to shift the emphasis from control to elimination.

**Figure 1:**
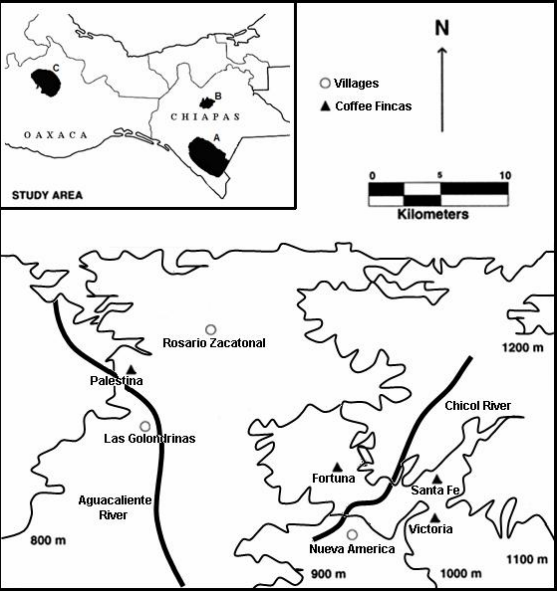
Map of the Mexican-Guatemalan border area showing the geographical location of the three villages and four coffee fincas studied within the southern Chiapas focus endemic for human onchocerciasis, Mexico (indicated by **A **in the inset).

In 1996, and before the present study was conducted, it had been assumed that the impact on transmission would be more evident in villages with high coverage of and compliance to ivermectin and nodulectomy; therefore three villages with such characteristics were selected for the study described here, namely: Las Golondrinas (92°39'17"W, 15°26'06"N, 920 m above sea level (masl)), Rosario Zacatonal (92°37'47"W, 15°27'25"N, 791 masl), and Nueva América (92°26'38"W, 15°17'08"N, 880 masl), which, prior to the introduction of ivermectin, had microfilarial prevalences of 69%, 79%, and 46% respectively [22]. Las Golondrinas and Rosario Zacatonal are 7.0 km apart from each other and a coffee finca (Palestina) is located between 2.5 and 3.0 km from each village respectively; they all constitute locality I. The village of Nueva América is surrounded by three coffee fincas, namely, La Victoria, La Fortuna, and Santa Fe, which are located at a distance between 2 and 5 km from the village; they all constitute locality II. The study area is depicted in Fig. 1. The closest simuliid breeding sites were located approximately at 6.0 km from Las Golondrinas, 1.0 km from Rosario Zacatonal, 2.5 km from Palestina, 2.0 km from Nueva América, 2.0 km from La Victoria, 1.0 km from La Fortuna, and 0.5 km from Santa Fe, i.e. within the flight range of *S. ochraceum *s.l. [23,24]. Treatment is typically administered to the eligible resident populations of each village in January and June of each year. In 1996, the average ivermectin treatment coverage among the total population had been of about 80%. However, for the purposes of this study, the residents were not treated as usual in January and June, but immediately after parasitological examination, which took place in May and November to coincide with the commencement of each of the annual coffee seasons (see below). A census of each community was conducted [10,16] at the start of each survey in 1997–1998 and 1998–1999, and eligible residents were treated just after the parasitological examination was completed.

### Migrant characteristics

The villages receive a biannual influx of temporary labourers (see below) who normally would be eligible for treatment but who, at the time of the study, were not on the records of the Mexican onchocerciasis elimination programme. Although the possibility cannot be excluded that the migrants might have been treated with ivermectin while at home (mainly in Guatemala, see below) it is also possible, and perhaps more likely, that they would have missed treatments at home because of being away in Mexico when treatment rounds were distributed. The temporary coffee workers would have therefore, been falling through the net in both countries.

Every year the cultivation of coffee includes two seasons, planting-clearing and harvesting. Planting-clearing is carried out from May through July and harvesting from November through February. The latter coincides roughly with the season during which onchocerciasis transmission has been deemed to be highest in neighbouring Guatemala [25]. Coffee fincas' owners or administrative personnel hire the coffee labourers. These field labourers arrive *en masse *(most of them from Guatemala) usually by trucks, buses, or on foot, before the start of each coffee season. After completing each task required during the corresponding season, the labourers leave the coffee fincas in the same manner. As the coffee labourers are usually not the same individuals from season to season, the name, place of residence abroad, and date of birth of each labourer was recorded on a registration form and confirmed by comparison with the payroll lists or their identification papers.

The coffee labourers were informed about the study and consent was requested and obtained before proceeding with skin snipping and parasitological examination. Most (> 80%) of the coffee labourers were adult males (≥ 18 years old), and more than 85% of the adult male population in each of the four coffee fincas agreed to participate in the study. The remainder (<20%) was made up of accompanying females and children but less than 10% of these participated in the study. Because migration patterns could change over time, both coffee cycles of 1997 to 1998 and of 1998 to 1999 were investigated in order to assess any variation in the migrant human and biting vector populations. A migrant was defined as a person who had worked in the coffee plantation for a whole season. In the following season, this person was most likely absent, but occasionally, he could participate in the study again if returning as a temporary worker to the area. Less than 10% of the migrants returned to the same fincas where they had worked before.

All examined migrant coffee labourers who were skin mf positive were offered ivermectin. Treatment was not given to those migrants with a severe medical condition, or those who refused to comply, and it was administered only after parasitological examination had been completed (see next section), or before returning home. In 1999, after the present study was completed and the migrant workers stopped receiving treatment, the Mexican onchocerciasis elimination programme was immediately notified. The programme continued treating biannually the residents in the three villages, but in 2003, it selected 37 out of 39 formerly hyperendemic villages, and 13 formerly mesoendemic villages in Southern Chiapas to receive three-monthly ivermectin treatment (4 rounds per year). Migrant labourers working on the coffee plantations are at present targeted for treatment.

### Parasitological studies

Using a corneoscleral (Holth type) 2 mm punch, four skin biopsies (two from the supra-scapular and two on the supra-iliac body regions) were taken from each participating individual in each village (residents) and coffee finca (migrants). Skin biopsies were incubated overnight in buffered saline, emerging mf counted and the snips weighed to estimate the intensity of infection per mg of skin [18]. The parasitological examinations of both residents and migrants were conducted before the migrants commenced their work, i.e. in May and November of 1997, and in May and November of 1998.

### Entomological studies

Entomological surveys to estimate onchocercal infection levels in host-seeking *S. ochraceum s.l*. were carried out in the three villages and the four coffee fincas. Flies were collected from May to August 1997, during the coffee planting-clearing season, and from November 1997 to February 1998, during the harvesting season. The entomological sampling was repeated in the coffee seasons of 1998–1999 following the same scheme.

*S. ochraceum s.l*. females were collected when landing on consenting volunteers from the villages, with each collection team composed of an attractant and a collector as previously described [26]. Daily entomological sampling, conducted for 15 days in four sites at each locality started at 07:00 hours and ended at 17:20 hours. Collections consisted of 20 min sampling units followed by 40 min breaks, and were conducted simultaneously in four sites for each community and coffee finca. All *S. ochraceum s.l*. specimens were dissected immediately after collection and during the rest period to determine parity [27]. The abdomen, thorax, and head of each parous female were examined for all stages of *O. volvulus *larvae, which were identified according to descriptive statistics [28].

### Data analysis

The point (crude) prevalence of skin infection with mf and the (arithmetic) mean number of mf/mg of skin were calculated for each study season and all examined residents in villages and migrant labourers in coffee fincas of localities I and II. Exact 95% confidence intervals were estimated for prevalence values [29]. In addition, the community microfilarial load (CMFL: the geometric mean number of mf per skin snip in those aged ≥ 20 years [30]) was also assessed for residents and migrants as the latter comprised mainly adults.

In onchocerciasis, and assuming random biting and lack of concentration of mf by the vector's saliva, the proportion of individuals who provide an infected blood meal to simuliid vectors has been approximated by the prevalence of skin-biopsy positives in the human population [31,32]. Based upon the specific *Onchocerca-Simulium *combination prevailing in the study area, the prevalence of infection in the thoraces of the vectors (which contain normally non-infectious L1 and L2 stages) is higher than the prevalence of infection in the heads (which contain only the infectious third stage larvae). Therefore, the prevalence of infection in the bodies of flies (heads plus thoraces) is the most sensitive indicator of parasite-vector contact, and thus it can be used effectively to monitor the presence of mf in untreated coffee fincas and villages subject to control. It has been proposed that assessment of the impact of any intervention on transmission be based on the total number of *O. volvulus *larvae (all stages) in bodies, and on detection of L3 in heads of flies [33]. Therefore, in the present study, the prevalence of infection was calculated as the number of flies positive for any *O. volvulus *larval stage divided by the total number of parous flies examined; the associated (exact) 95% confidence intervals (95% CI) were determined [29], and the results were expressed per 1,000 parous flies [34].

Differences between proportions of infected flies between coffee seasons for each site and for each year, as well as before arrival (April and October), during the stay (May through July, and November to February), and after departure (August and February) of migrants in localities I and II were tested by means of the (one-tailed) Fisher's exact modification of the 2 × 2 chi-squared test [35]. A *p*-value < 0.05 indicated statistical significance.

## Results

### Infection in migrants and resident populations

As shown in Table 1, the total number of migrants in coffee fincas for each season in 1997–1998 and 1998–1999 varied widely (from 35 to 850 individuals). In contrast, the numbers of the resident population ranged from 350 to 437 individuals. Thus, the ratio of migrants to residents varied from 0.1:1 in locality I during the planting season of 1997–1998 and that of harvesting in 1998–1999, to 2:1 in locality II during the harvesting season of 1997–1998, and that of planting-clearing of 1998 (Table 1). Overall, during the whole study 57% of the migrants came from the Guatemalan Department of Huehuetenango (North-western hypoendemic focus), while the remaining 43% came from the non-endemic Guatemalan Departments of San Marcos, and Petén. Only 0.01% came from other endemic areas of Southern Mexico. The migrant population working in the fincas during each coffee season mainly comprised newly hired people. The proportion of residents that were skin snipped ranged from 51 to 65% for locality I, and from 17 to 63% for locality II, whilst the equivalent proportions for migrants varied between 37 and 67%, and between 11 and 88%. There were infected migrants in all coffee fincas, their microfilarial prevalence ranging from 3% (95% CI = 2–6%) in locality II at the start (November) of the harvesting season of 1997–1998 (when they represented 71% of the total population at the time), to 23% (10–42%) in locality I at the beginning (May) of the planting season of 1997 (when they represented 9% of the total population). CMFL values for the migrants ranged from 0.03 to 0.71 mf/snip. The prevalence in the resident population of the villages in locality I ranged from 13% (9–18%) in 1997 to 4% (95% CI = 2–8%) in 1998, but apart from these values, none of the other figures were significantly different. In Las Golondrinas, treatment coverage ranged between 80 and 88% of the population. In locality II microfilarial prevalence varied between 14% (10–20%) in 1997 and 7% (2–16%) in 1998. In general the prevalence of infection in the migrant population was higher in locality I than in locality II, but this difference was significant only for the planting season of 1997, and the ratio of migrants to residents was higher in locality II than in locality I.

**Table 1:** Population (number examined), number positive for *Onchocerca volvulus *mf [prevalence], and mean microfilaridermia (arithmetic mean no. of mf/mg and community microfilarial load) at beginning of coffee planting-clearing and harvesting seasons of 1997–1998 and 1998–1999 in localities I (villages of Golondrinas, Rosario Zacatonal, and finca Palestina), and II (village of Nueva América, and fincas of Victoria, Fortuna, and Santa Fé) in Southern Chiapas, Mexico

**Localities**	**Residents**			**Migrants**			**Ratio of migrants to residents**
		
	**Population (Examined)**	**Pos [Prev] (95% CI)**	**AM mf/mg (CMFL)**	**Population (Examined)**	**Pos [Prev] (95% CI)**	**AM mf/mg (CMFL)**	
**Planting season (May-July 1997)**							
Locality I	437 (318)	36 [11.3%] (8.1–15.3)	0.28 (0.19)	45 (30)	7 [23.3%] (9.9–42.3)	0.78 (0.71)	0.1 : 1
Locality II	374 (235)	34 [14.4%] (10.2–19.6)	1.27 (0.51)	350 (204)	12 [5.9%] (3.1–10.0)	0.12 (0.08)	0.9 : 1
**Harvesting season (November 1997–February 1998)**							
Locality I	385 (250)	32 [12.8%] (8.9–17.6)	0.25 (0.14)	130 (48)	3 [6.3%] (1.3–17.2)	0.54 (0.23)	0.3: 1
Locality II	350 (198)	15 [7.6%] (4.3–12.2)	0.53 (0.22)	850 (258)	7 [2.7%] (1.1–5.5)	0.07 (0.03)	2.4 : 1
**Planting season (May-July 1998)**							
Locality I	370 (236)	30 [12.7%] (8.7–17.6)	0.18 (0.11)	N/A	N/A	N/A	N/A
Locality II	350 (61)	4 [6.6%] (1.8–15.9)	0.06 (0.05)	600 (68)	6 [8.8%] (3.3–18.2)	0.22 (0.09)	1.7 : 1
**Harvesting season (November 1998–February 1999)**							
Locality I	370 (187)	7 [3.7%] (1.5–7.6)	0.11 (0.06)	35 (14)	2 [14.3%] (1.8–42.8)	1.58 (0.39)	0.1 : 1
Locality II	N/A	N/A	N/A	190 (168)	9 [5.4%] (2.5–9.9)	0.26 (0.12)	N/A

N/A: Data not available.

### Infection in vectors

A total of 28,999 and 25,823 *S. ochraceum s.l*. females were collected, respectively in villages and fincas during the study (Table 3). The entomological data are summarized by locality in Table 2. The percentage of parous flies was significantly higher in locality I, 73.8% (95% CI = 73.3–74.2%) than in locality II, 68.2% (67.5–68.8%). However, the number of flies harbouring *O. volvulus *larvae of any stage per 1,000 parous flies was significantly lower in locality I, 0.8 (0.5–1.2) than in locality II, 2.3 (1.5–3.2).

**Table 2:** Total number of parous and examined *Simulium ochraceum s.l*., the percentage of parous flies, the number of infected flies, and the prevalence of infection (with any *Onchocerca volvulus *larval stage) per 1,000 parous flies at localities I (including the villages of Las Golondrinas, Rosario Zacatonal, and finca Palestina), and II (including the village of Nueva América, and fincas Victoria, Fortuna, and Santa Fé) in the Southern Chiapas focus, Mexico

**Locality**	**N° of flies parous/examined (% parous)**	**No of infected flies with any larval stage**	**No of infected per 1,000 parous flies (95% CI)**
Locality I	26,068/35,336 (73.8)	20	0.8 (0.5–1.2)
Locality II	13,284/19,486 (68.2)	30	2.3 (1.5–3.2)

**Table 3:** Total number of parous and examined *Simulium ochraceum s.l*., the percentage of parous flies, the number of infected flies, and the prevalence of infection (with any *Onchocerca volvulus *larval stage) per 1,000 parous flies detailed by village and coffee finca for each of the study in the Southern Chiapas focus, Mexico

**Locality Village Coffee finca**	**N° of flies parous/examined (% parous)**	**No. of infected flies No. per 1,000 parous flies (95% CI)**	**N° of flies parous/examined (% parous)**	**No. of infected flies No. per 1,000 parous flies (95% CI)**
	**Planting-clearing season of May-Jul 1997**		**Harvesting season of Nov 1997-Feb 1998**	
Locality I				
Las Golondrinas	2,636/3,017 (87.4)	5 1.9^§ ^(0.6–4.4)	2,055/2,636 (78.0)	0 0^§ ^(0–1.8)
Rosario Zacatonal	2,788/3,384 (82.4)	2 0.7 (0.1–2.6)	2,907/3,947 (73.7)	2 0.7 (0.1–2.5)
Finca Palestina	3,538/4,115 (86.0)	7 2.0^a,c ^(0.8–4.1)	4,834/6,786 (71.2)	1 0.2^a,d ^(0–1.2)
Locality II				
Nueva América	828/956 (86.6)	2 2.4 (0.3–8.7)	967/1,534 (63.0)	1 1.0 (0–5.7)
Fincas Victoria, Santa Fé and Fortuna	555/625 (88.8)	5 9.0^b,c ^(2.9–20.9)	2,540/3,341 (76.0)	5 2.0^b,d ^(0.6–4.6)
	**Planting-clearing season of May-Jul 1998**		**Harvesting season of Nov 1998-Feb 1999**	
Locality I				
Las Golondrinas	1,483/2,098 (70.7)	0 0 (0–2.5)	2,591/3,983 (65.1)	0 (0–1.4)
Rosario Zacatonal	548/874 (62.7)	0 (0–6.7)	384/581 (66.1)	1 2.6 (0.1–14.4)
Finca Palestina	N/A	N/A	2,304/3,915 (58.9)	2 0.9 (0.1–3.1)
Locality II				
Nueva América	681/1,032 (66.0)	4 5.9^§ ^(1.6–15.0)	3,254/4,957 (65.6)	5 1.5^§ ^(0.5–3.6)
Fincas Victoria, Santa Fé and Fortuna	561/693 (81.0)	1 1.8 (0–9.9)	3,898/6,348 (61.4)	8 2.1 (0.9–4.0)

^a, b, c, d ^Denote statistical significance (*p *< 0.05) with the same letter indicating the comparison under scrutiny.

^§ ^Indicates borderline significance (*p *= 0.05).

Table 3 disaggregates the entomological data by site and coffee season. For reasons of sample size the data for the three fincas of locality II (namely, Victoria, Santa Fé and Fortuna) are combined. During the years 1997–1998, the proportion of parous flies was significantly higher for all sites (all *p*-values << 0.01) in the planting-clearing season (May through July) than in the harvesting season (November through February). Accordingly, the number of infected *S.ochraceum s.l*. per 1,000 parous flies tended also to be higher during the former than during the latter coffee season, but this was statistically significant only in Finca Palestina (*p *= 0.012) of locality I and Fincas Victoria, Santa Fé, and Fortuna combined (*p *= 0.021) of locality II; the values in the villages of Las Golondrinas and Rosario Zacatonal are suggestive of the same trend but only of borderline significance (*p *= 0.05). The proportion of infected flies in the coffee fincas of locality II was significantly higher than that of the finca in locality I (*p *= 0.02) during May-July 1997, and also (*p *= 0.02) during November 1997–February 1998. Due to lack of entomological data for one of the fincas during the planting season of 1998 a similar comparison was not possible, and although suggestive of the same trend, the difference for the harvesting season of 1998–1999 was not significant

**Figure 2:**
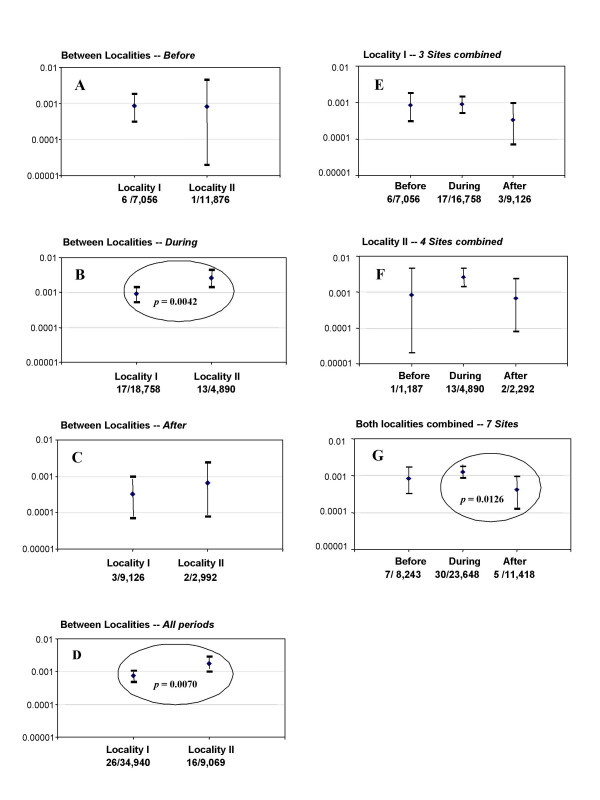
The prevalence of infection (with any *Onchocerca volvulus *larval stage) in *Simulium ochraceum s.l*. parous flies in localities I and II: **A**, *before *the arrival (April and October), **B**, *during *the stay (May through July and November through February), **C**, *after *departure (August and February) of temporary migrant workers in the coffee seasons of 1997–1998 and 1998–1999 in the Southern Chiapas onchocerciasis focus, Mexico. **D **compares localities I and II for all three periods combined; **E **and **F **compare, respectively within localities I and II, fly infection levels between the *before*, *during*, and *after *periods, and **G **compares the periods for both localities combined. Error bars denote 95% confidence intervals; ovals indicate statistical differences with associated *p*-values.

The proportion of infected parous *S. ochraceum s.l*. flies in population samples was also analyzed within coffee seasons, i.e., *before *the arrival of migrants (April and October), *during *the stay of migrants (planting-clearing in May through July, and harvesting in November through February), and *after *departure of migrants (August and February), and compared both between and within localities I and II. The data are summarized in Fig. 2. There was a significant difference (*p *= 0.0069) between proportions of positive flies between localities when all time periods were combined (Fig. 2D), with locality II exhibiting a higher infection rate (see also Table 2). This was attributable to the higher prevalence of flies with *O. volvulus *larvae found *during *the stay of the temporary workers (Fig. 2B). Overall there was a significant difference (*p *= 0.0126) between the *during *and *afte*r periods (Fig. 2G), with the proportion of flies infected *during *the stay of the temporary workers being higher than that *after *their departure. Although the proportion of positive flies *during *the stay period was higher than that in the *before *period (particularly for locality II), the difference was not significant (*p *= 0.45). The proportions of positive flies *after *departure of the workers were always lower than those *during *their stay (Figs. 2E for locality I and 2F for locality II), but this difference was statistically significant (*p *= 0.0126) only when all sites were combined (Fig. 2G).

## Discussion

The effect of migration on onchocerciasis transmission has long been recognised in Guatemala [23], where indigenous peoples from non-endemic, northern areas make annual visits to the Yepocapa focus during the coffee-harvesting season and become infected with onchocerciasis [36]. In Ecuador, peripheral endemic foci have also been formed by migration of infected populations from the main Santiago focus [37]. This migration is a serious concern because despite multiple treatments with ivermectin, transmission persists in the Río Capayas of Ecuador, and this is likely to be partly due to significant migration of potentially infected individuals from hyperendemic communities along the Río Cayapas to the Río Santiago and vice versa [13]. The risk that migration of infected individuals to non-endemic areas with susceptible and anthropophagic simuliid species could contribute to the establishment of new foci has also been explored entomologically in Venezuela [38] and more extensively in Brazil [2].

In Mexico, and after seven years of biannual administration of ivermectin, the prevalence and intensity of microfilarial infection were substantially reduced in Las Golondrinas, its status changing from hyper- to hypoendemic [16]. However, the fact that children born after the start of ivermectin distribution presented with anti-*O. volvulus *antibodies as well as with skin mf [10], suggested that transmission was ongoing in the Southern Chiapas focus. Recent entomological surveys carried out during 2001 in several villages of this focus reported the occurrence of infected and infective *S. ochraceum s.l*. in the host-seeking vector population, confirming that interruption of transmission has not yet been accomplished [39,40]. A regular influx of untreated and potentially infected temporary coffee labourers could provide important sources of microfilarial infection to local vectors, helping to perpetuate transmission.

At short distances from the villages included in this study, there were a number of coffee fincas which receive an itinerant population of migrant labourers twice yearly. In addition, these temporary workers are more likely than not to have been excluded by ivermectin distribution campaigns both in their locality of provenance and in the coffee plantations. In this study, prevalence of microfilarial infection among workers ranged from 3 to 23% (from 4 to 14% among residents), and in locality II the number of migrants more than doubled that of residents at the time of their presence. Thus, although treatment coverage in the villages may be above 80%, overall coverage in the area may have at least been halved. In a study in Sierra Leone, five doses of ivermectin given every six months with a coverage level of about 30% did not affect transmission potentials of *S.damnosum s.l*. [41], but this species has a higher vector competence than *S. ochraceum *at low skin microfilarial levels [42]. In West Africa, the prevalence of mf was considerably higher in migrants from outside the Onchocerciasis Control Programme area than in its residents because the latter had been protected through vector control for over 14 years [43,44].

Overall, the proportion of infected flies was significantly lower in locality I (where the ratio of migrants to residents had ranged from 0.1 to 0.3:1) than in locality II (where it ranged from 1 to 2.4:1), although parity was significantly higher in flies collected at locality I. Also, when the contingent of untreated persons entered the coffee fincas, fly infection levels increased. There was evidence that the departure of migrants was associated with a fall in the proportion of flies harbouring *O. volvulus *larvae, but due to the low numbers of flies infected, among other things (see below), it is not possible to claim that this association is truly causal.

A complication of the study design was the need, for ethical reasons, to treat the migrants that consented to participate following parasitological examination; to try and treat them all before departure would not have been always possible as they may leave in a staggered fashion and the researchers may not have been in the fincas at the time. However, in locality II, as few as 11% of migrants were examined and therefore treated (planting season of 1998, when they represented >60% of the total population). Also, a number of labourers would have arrived and started working in the plantations before the entrance of the research team. During the study, treatment coverage levels among migrants would have been considerably lower than those among residents. It is important also to realize that temporary labourers work from dawn to dusk and use very little protection in terms of clothing when in the fields, exposing themselves maximally to the bites of the local simuliids.

The number of total *S. ochraceum s.l*. flies collected in the villages (ca. 29,000 flies) was very similar to that collected in the coffee fincas (ca. 26,000 flies), and breeding sites were probably very close to the places of habitation and work, i.e., within the flight range (~10 km) of the local vectors [23,24], as indicated by the high proportion of parous flies in both villages (73%; 95% CI = 72–74%) and fincas (68%; 95% CI = 67–69%), suggesting that both places were included within the foraging area of *S.ochraceum *s.l. populations [24]. Interestingly, the proportions of parous and infected flies were higher during the planting (May through July) than during the harvesting (November through February) seasons, contrary to the expectation of older flies in the host-seeking population and more intense transmission during the latter that stems from studies conducted in Guatemala in the late 1980's/early 1990's, i.e., nearly 20 years ago. Admittedly, in the present study analyses were not conducted by onchocercal larval stage as the interest was to ascertain the level of general infection in the flies with emphasis on the transmission from humans to vectors; also the statistical analyses by infection stage for the periods before the arrival, during the stay, and after the departure of the coffee workers would have lost power. A previous study [33] of parity and infection rates conducted during 1990–1991, prior to the introduction of mass ivermectin distribution in Las Golondrinas, had reported a significantly higher proportion of parous flies in the coffee areas surrounding the village than in the village itself. Roughly, 16 infected flies per 1,000 parous were found for both May-June and November-February (with a mean of ~0.05 larvae per parous fly for both periods), suggesting that seasonality of transmission may less marked in Mexico or that its pattern may differ from that in Guatemala. Also, the parous rates seem to have increased from those ranging between 25% (in September 1990) and 54% (in June 1991) before wide ivermectin administration, to those ranging from 65% (Nov 1998–Feb 1999) to 87% (May-Jul 1997), after 10–13 treatment rounds, suggesting that the question of whether or not a reduced microfilarial reservoir in the human population may result in increased vector survival should be explored. This issue has been investigated through fly-feeding experiments [45] but scarcely in natural populations. The seasonality in age-composition and infection/infectivity of *S.ochraceum s.l*. host-seeking populations before and after mass drug administration should be studied in the onchocerciasis endemic areas of southern Mexico given the availability of long-term studies in Chiapas [[4,11,16,19,21,22,26,33,38], and [39]].

The data indicate that the arrival of temporary infected labourers in and out the southern Chiapas focus, and the resulting failure for them to receive regular treatment may maintain the transmission of the parasite from humans to the abundant local vector populations. It would also be necessary to evaluate transmission from vectors back to humans in order to ascertain the true potential for onchocerciasis transmission due to the temporary presence of migrants. In the present study the rates of infectivity (with L3 larvae) were very low, precluding analyses of infection by larval stage. Although we cannot demonstrate conclusively that the arrival of coffee labourers truly increases the intensity of transmission in areas under regular treatment, the results are suggestive that their presence may indeed increase the levels of vector infection.

Although the level of ivermectin coverage and the duration of treatment programmes that are necessary to halt transmission in the area have not yet been determined, it is clear that a coverage lower than 85% for ~20 (biannual) rounds may be insufficient for this purpose [39,40] even in the presence of a relatively poor vector host such as *S. ochraceum s.l*. [15]. In 2003 the biannual treatment strategy was modified in the majority of the formerly hyperendemic communities of this focus by increasing treatment frequency to four times per year in order to attain higher coverage and accelerate interruption of transmission. This new strategy also includes treating all temporary coffee workers. Currently however, and due to the worldwide fall in coffee price, migration of coffee labourers in and out the southern Chiapas focus has decreased considerably. Additional epidemiological studies will be conducted to evaluate the impact of the new strategy on parasite transmission. Sampling protocols for the accompanying entomological monitoring may need to be revised.

## Competing interests

The author(s) declare that they have no competing interests.

## Authors' contributions

MARP designed and supervised the project, analyzed data, and drafted the manuscript.

ASC and CLO prepared the databases for analyses and assisted in typing the manuscript.

M-GB checked the databases, performed analyses, and helped write the final version of the manuscript.

JBD helped draft the manuscript, proposed and performed some of the statistical analyses, and reviewed the final version of the manuscript.

All authors approved the paper.
